# Supplementary material for: NEK2 Phosphorylates RhoGDI1 to Promote Cell Proliferation, Migration and Invasion Through the Activation of RhoA and Rac1 in Colon Cancer Cells
Source: Cells. 2024 Dec 16;13(24):2072. doi: 10.3390/cells13242072 (PMC11674122; doi:10.3390/cells13242072)
Supplement: Supplementary file 1 [file cells-13-02072-s001.zip › cells-3204362-supplementary.pdf]

Supplementary figures

A

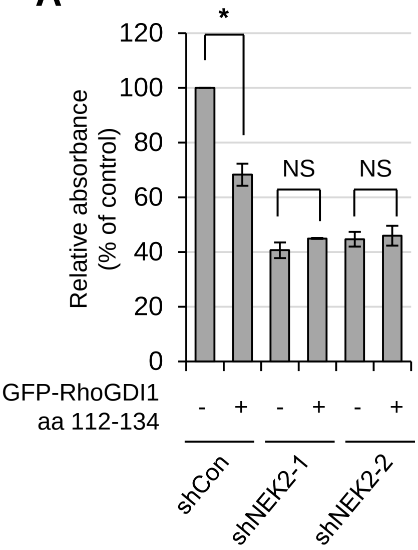

B

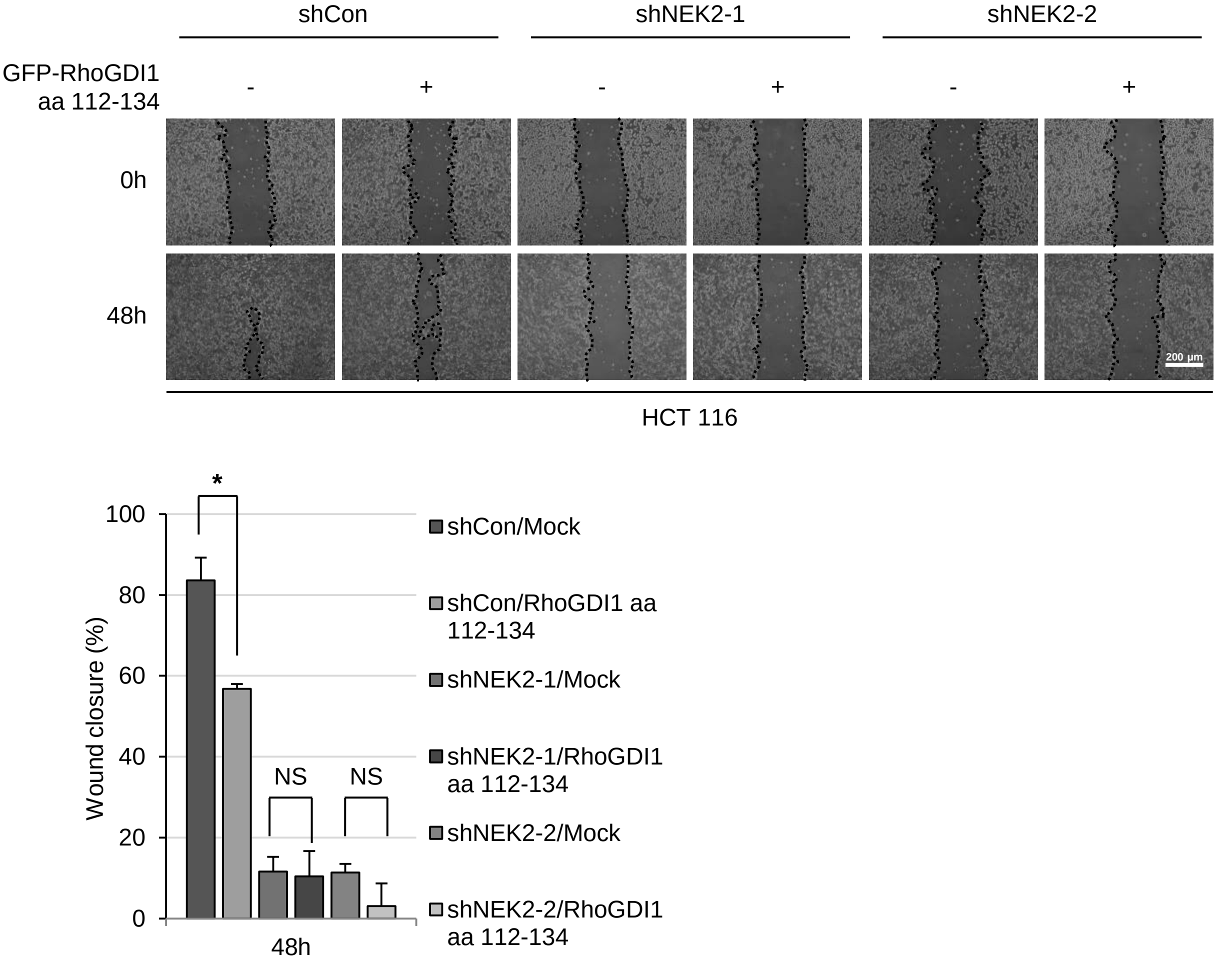

Supplementary figures

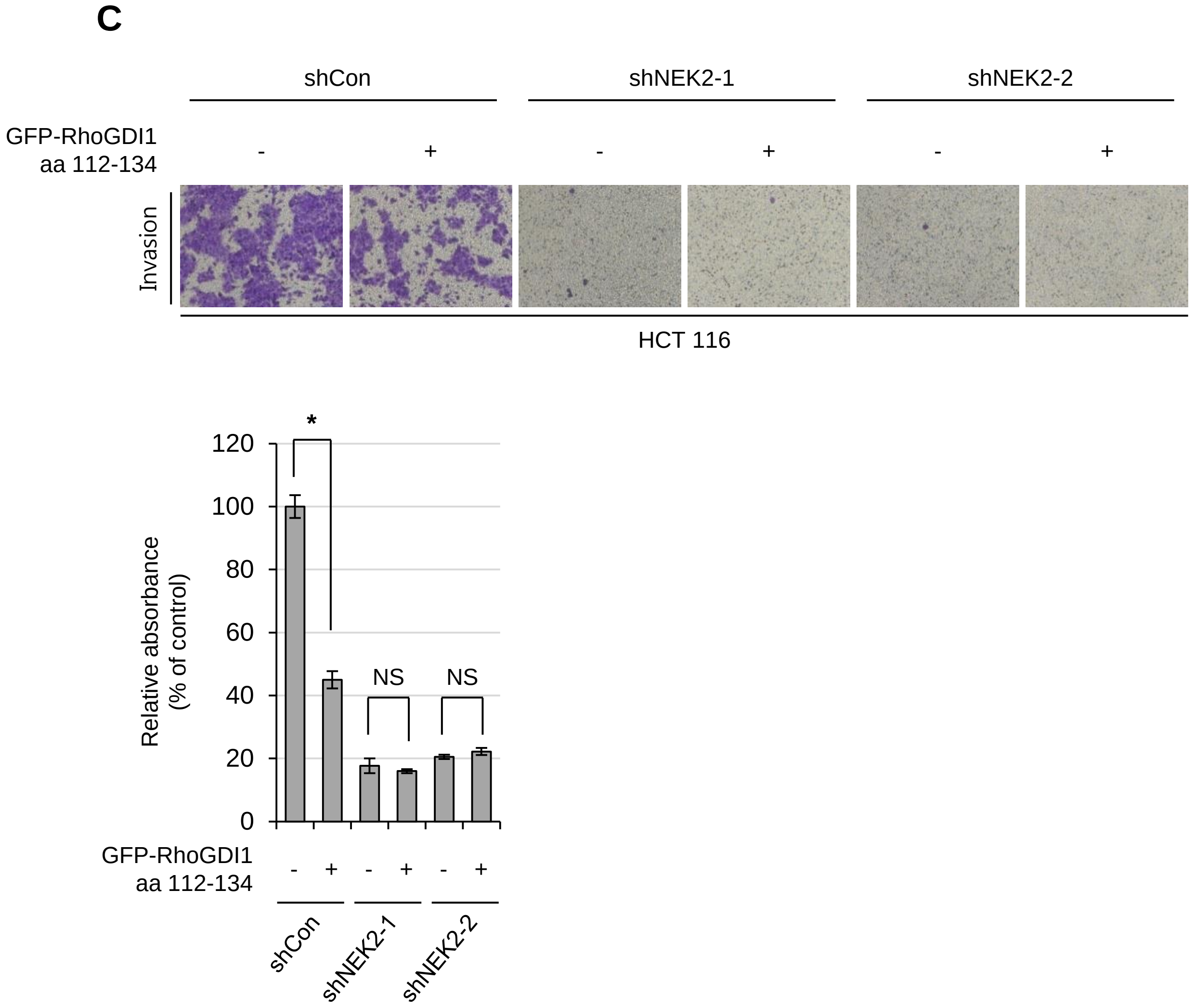

Interaction of NEK2 with aa 112-134 on RhoGDI1 is crucial for promoting proliferation, migration, and invasion of colon cancer cells. HCT 116 cells expressing control shRNA or two NEK2 shRNAs were transiently transfected GFP or GFP-RhoGDI1 aa 112-134. (**A**) Cells were subjected to WST-8 assay. The graph represents the relative percentages of proliferating cells. (**B**) Migration of indicated cells was evaluated by wound healing at each time point. Representative images of migrating cells obtained at 48 h after wound formation (left). Scale bar=200 μm. The migration was quantified by calculating the cell-covered area using Image J (right). (**C**) Cells were incubated in serum-free media for 24 h and then subjected to transwell invasion assay. Representative images of invading cells are displayed on the left, and the relative percentages of invasion are quantified on the right. Quantitative data represent the mean ± S.D. (n=3). *P<0.05; **P<0.01.
